# Rhythmic auditory stimulation combined with split-belt treadmill training improves spatiotemporal gait parameters in hemiparetic stroke patients: a randomized controlled trial

**DOI:** 10.3389/fneur.2026.1904812

**Published:** 2026-10-14

**Authors:** Minghui Du, Wei Zhang, Liang Xia, Xiaowu Xia, Chenglin Qiu, Yixin Zhan, Zhiyuan Zhang, Longwei Chen

**Affiliations:** 1 School of Physical Education, Xi’an Jiaotong University, Xi’an, Shaanxi, China; 2 Lizhi College, Xi’an Jiaotong University, Xi’an, Shaanxi, China; 3 Human Resources Department, Xi’an Jiaotong University, Xi’an, Shaanxi, China; 4 Rehabilitation Science Institute, Shaanxi Provincial Rehabilitation Hospital, Xi’an, Shaanxi, China

**Keywords:** gait parameters, post-stroke hemiparesis, randomized controlled trial, rhythmic auditory stimulation, split-belt treadmill training

## Abstract

**Background:**

Split-belt walking training (SWT) primarily ameliorates spatial gait asymmetry after stroke, yet its effect on temporal gait parameters remains limited. Rhythmic auditory stimulation (RAS) enhances cadence and rhythmicity via auditory–motor coupling. This study examined whether combining RAS with SWT yields greater walking benefits than SWT alone or conventional rehabilitation.

**Methods:**

Thirty patients with post-stroke hemiparesis were randomized to RAS+SWT (*n* = 11), SWT alone (*n* = 8), or conventional rehabilitation (control, *n* = 11). Interventions were delivered for three weeks (five sessions/week). Primary outcomes were spatiotemporal gait parameters: walking speed, cadence, step length, and limping index. Linear mixed-effects models were used, with Hedges’ g for effect sizes.

**Results:**

No dropouts occurred. Significant time-group interactions were found for walking speed (*F* = 10.68, *p* < 0.001), cadence (*F* = 5.01, *p* = 0.014), and affected-side step length (*F* = 6.21, *p* = 0.006). The RAS+SWT group showed greater improvements than controls in walking speed (mean difference = 0.167, 95% CI [0.090, 0.245]), cadence (mean difference = 9.340, 95% CI [4.456, 14.223]), and step length (unaffected: mean difference = 0.077, 95% CI [0.039, 0.116]; affected: mean difference = 0.081, 95% CI [0.035, 0.127]), and greater walking speed improvements than the SWT group (mean difference = 0.110, 95% CI [0.022, 0.197]). For the limping index, a significant main effect of group was observed (*F* = 5.41, *p* = 0.011); however, the time-by-group interaction was not significant (*F* = 0.430, *p* = 0.655).

**Conclusion:**

RAS combined with SWT is more effective than conventional rehabilitation in improving multidimensional spatiotemporal gait parameters—including walking speed, cadence, and affected-side step length—in patients with post-stroke hemiparesis. Compared with SWT alone, the combined intervention yielded greater improvements in walking speed, while no significant between-group differences were observed for cadence or step length. The combined protocol is feasible and safe.

**Clinical trial registration:**

ChiCTR2600121963.

## 1 Introduction

Stroke is one of the leading causes of severe long-term disability in adults worldwide (1), with over 60% of acute survivors experiencing varying degrees of motor impairment (2). Among these impairments, hemiparetic gait dysfunction is particularly prominent (3). It not only severely restricts patients’ ability to participate in community activities but is also closely associated with an elevated risk of falls, diminished quality of life, and increased caregiver burden (4, 5). Accordingly, gait restoration is widely recognized as a core objective in stroke rehabilitation. However, even after conventional rehabilitation, a considerable proportion of patients continue to exhibit substantial gait deficits (6), among which abnormalities in spatiotemporal gait parameters—particularly bilateral asymmetry—are the most persistent (7). Specifically, individuals with hemiparesis often present with reduced stance time on the affected side, a compensatory increase in step length on the unaffected side, and an overall reduction in walking speed (7). This aberrant pattern further exacerbates energy expenditure and limits functional mobility (7).

To address these gait deficits in a targeted manner, researchers have developed a range of rehabilitation techniques grounded in motor learning principles. Among these, split-belt treadmill training (SWT) operates by driving the two belts at different speeds, thereby artificially inducing movement errors and triggering sensorimotor adaptation processes (8, 9). A substantial body of evidence indicates that SWT can effectively ameliorate spatial gait asymmetry after stroke, and these improvements can be partially retained during overground walking once the perturbation is removed—a phenomenon referred to as the after-effect (8). However, the primary therapeutic target of SWT is the spatial dimension of gait (10), and its effects on optimizing gait rhythmicity, cadence, and bilateral temporal coordination remain relatively limited (11, 12). Complementing this limitation, rhythmic auditory stimulation (RAS) provides a stable external temporal cue that drives synchronization between the auditory and motor systems, thereby significantly improving cadence, gait rhythmicity, and temporal symmetry (13). Mechanistically, RAS is thought to reduce the perceptual load of walking control, facilitating a shift from reliance on impaired proprioceptive feedback to a rhythm-guided motor control mode (14). Therefore, strategically integrating SWT, which targets spatial adaptation, with RAS, which targets temporal rhythmicity, may theoretically enable multidimensional and combined improvement of hemiparetic gait after stroke.

Although the mechanistic complementarity described above is theoretically compelling, clinical evidence regarding the combination of RAS and SWT remains limited. Existing studies have primarily focused on the combined application of RAS with conventional treadmill training, and findings suggest that RAS can yield additional gains in walking speed and cadence (15); however, its effects on improving gait asymmetry have been inconsistent across studies (16, 17). Meanwhile, SWT research has largely concentrated on its isolated effects, and investigations exploring whether the synchronous introduction of rhythmic cues during training can influence the adaptation process remain insufficient. More importantly, previous intervention studies have commonly adopted walking speed as the primary outcome measure (18, 19), whereas multidimensional analysis of gait parameters—particularly aspects of gait control such as rhythmicity and symmetry—has received comparatively little attention. This knowledge gap limits our understanding of the true effects of combined interventions and hinders the design of individualized rehabilitation programs tailored to patients with distinct gait deficits.

Against this background, the present study employed a randomized controlled trial design to systematically evaluate the effects of RAS combined with SWT, compared with SWT alone and conventional rehabilitation, on walking capacity in patients with post-stroke hemiparesis. We hypothesized that the combined intervention of RAS and SWT would produce additive effects, yielding greater improvements in multidimensional spatiotemporal parameters—including walking speed, cadence, affected-side step length, and gait symmetry—relative to SWT alone. The findings of this study are expected to provide evidence-based support for optimizing combined intervention strategies in post-stroke gait rehabilitation and to offer new insights into the interplay of distinct motor learning mechanisms underlying locomotor recovery.

## 2 Methods

### 2.1 Study design

This study was a prospective, single-blind, randomized controlled trial. The study protocol was approved by the Ethics Committee of Shaanxi Provincial Rehabilitation Hospital and registered with the Chinese Clinical Trial Registry (registration number: ChiCTR2600121963). Written informed consent was obtained from all participants prior to enrollment. The trial was conducted in strict accordance with the ethical standards of the Declaration of Helsinki.

### 2.2 Participants

The participants in this study were patients with post-stroke hemiparesis who were hospitalized at Shaanxi Provincial Rehabilitation Hospital between January 2026 and April 2026.

Inclusion criteria: (1) Diagnosis of stroke in accordance with the diagnostic criteria established by the Chinese Medical Association Neurology Branch, confirmed by CT or MRI imaging; (2) Clinically stable with stable vital signs, and no cognitive impairment or contraindications to exercise training; (3) Aged 25 to 75 years; (4) Time since stroke onset greater than one month; (5) Modified Rankin Scale score of 3 to 5; (6) Functional Ambulation Category (FAC) score below 2; (7) Basic comprehension of the assessment and training procedures, with a compliance rating (S5Q) ≥ 3; (8) Cardiac rehabilitation risk stratification classified as low or moderate risk.

Exclusion criteria: (1) Presence of unstable cardiac conditions, such as severe arrhythmia or valvular dysfunction; (2) Poorly controlled blood pressure at rest (>180/100 mmHg); (3) Subarachnoid hemorrhage; (4) Severe speech, visual, auditory, or psychiatric disorders affecting cognitive function; (5) Pregnant or breastfeeding women; (6) Concurrent participation in another interventional clinical study.

It should be noted that all enrolled participants had a FAC score of 1 according to our institutional assessment. The FAC assessment in our center tends to be conservative, and the enrolled participants represented a subgroup with relatively preserved functional walking ability, as reflected by their modified Barthel Index (75–81) and standing balance grade (2–3). Importantly, FAC < 2 was not the sole inclusion criterion; all participants also met the other inclusion criteria, including sufficient cognitive function (S5Q ≥ 3), stable medical status, and low-to-moderate cardiac risk. In addition, although the inclusion criteria specified an mRS score of 3 to 5, all participants who were ultimately enrolled had an mRS score of 3 to 4.

Participants meeting any of the following conditions during the study were excluded or classified as withdrawals: poor adherence during the intervention period rendering them unable to complete the prescribed training regimen; occurrence of serious adverse events or new-onset conditions such as recurrent cerebral hemorrhage making continuation in the trial inappropriate; voluntary withdrawal by the participant; or failure to complete the required number of rehabilitation sessions for any reason. All excluded and withdrawn cases were documented by designated personnel with reasons specified, ensuring the traceability of the study procedures.

### 2.3 Sample size estimation

Sample size was calculated using G*Power software (version 3.1.9.7). A repeated-measures ANOVA with within-between interaction (2 time points × 3 groups) was used as the analytical framework. Assuming a moderate-to-large effect size (Cohen’s *f* = 0.30), a correlation of 0.5 between repeated measures, α = 0.05, and power = 0.80, the estimated total sample size was 30 participants (approximately 10 per group). Due to single-center inpatient constraints, the maximum feasible sample size was 30 participants. No dropout occurred, and the final sample of 30 participants met the *a priori* requirement.

### 2.4 Randomization and allocation

Participants were randomly assigned to the RAS combined with SWT group (*n* = 11), the SWT group (*n* = 8), or the control group (*n* = 11). The random allocation sequence was generated by an independent statistician not involved in intervention delivery or outcome assessment using Excel software. Random numbers were assigned sequentially according to the order of participant enrollment, and participants were allocated to the respective groups based on predefined random number intervals. Group assignment information was recorded on cards, which were then sealed in opaque envelopes labeled only with the participant enrollment number on the outside. The envelopes were kept by the statistician and were opened only after participants had signed the informed consent form, at which point the group assignment was disclosed.

Owing to the nature of the interventions (the RAS+SWT group required training on a split-belt treadmill while wearing auditory stimulation devices, the SWT group trained on the split-belt treadmill only, and the control group received conventional rehabilitation), blinding of participants and the therapists delivering the interventions was not feasible. In this study, researchers who were blinded to group assignment were responsible for all gait data collection and analysis. During the data analysis phase, statisticians had access only to the coded, anonymized dataset, thereby minimizing assessment bias.

### 2.5 Intervention protocol

All three groups received conventional rehabilitation throughout the study period, including manual therapy and active limb exercises. In addition, the RAS+SWT and SWT groups received a 25-min treadmill intervention for three weeks, with a training frequency of five sessions per week (Figure 1).

**Figure 1:**
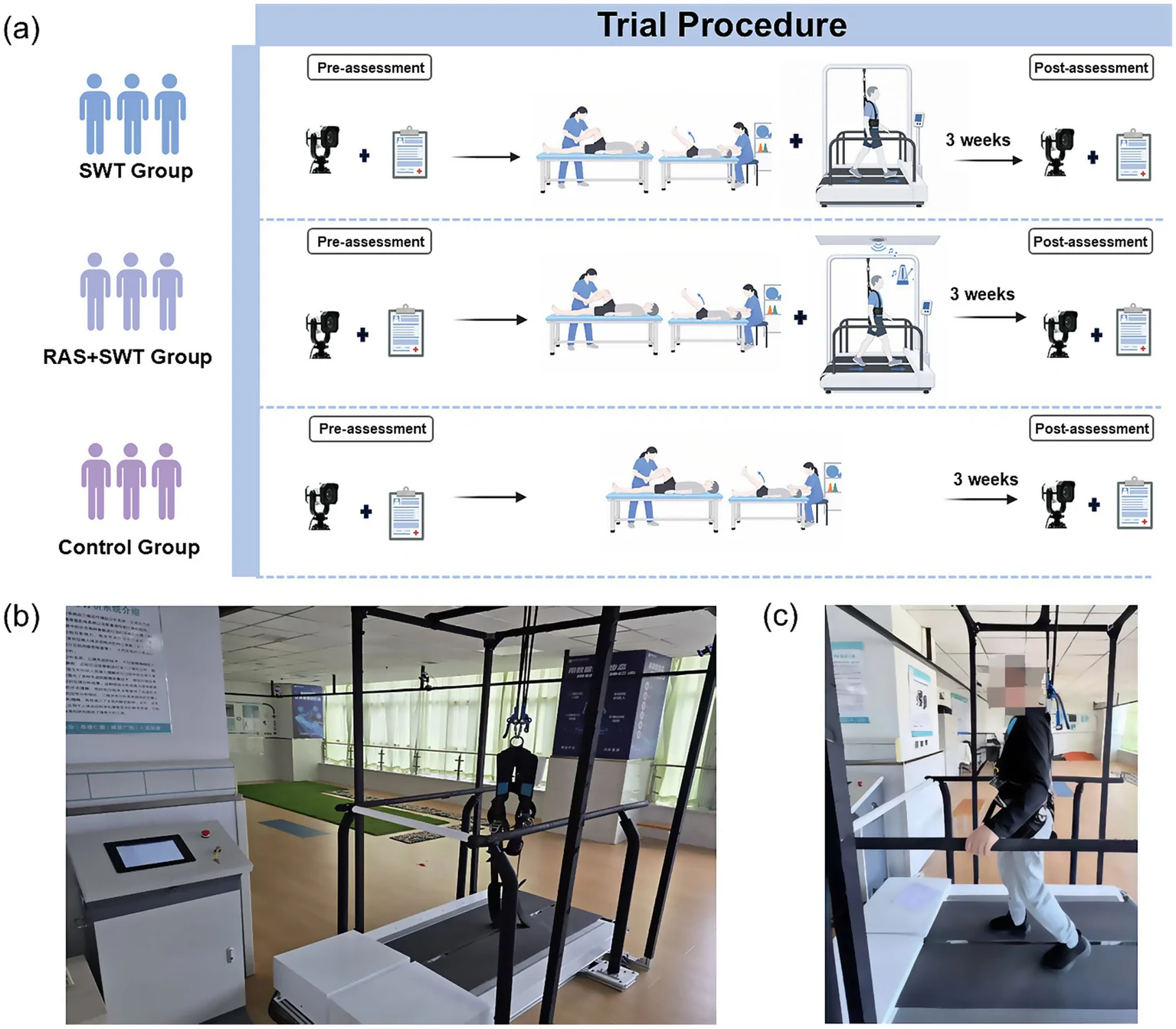
Overview of the intervention protocol and split-belt treadmill setup. **(a)** Schematic diagram of the intervention protocol for the three groups. **(b)** Photograph of the split-belt treadmill (SWT) system used in this study. **(c)** Photograph of a participant walking on the split-belt treadmill during training. The participant’s face has been obscured to protect privacy. SWT, split-belt walking training; RAS, rhythmic auditory stimulation; RAS+SWT, combined rhythmic auditory stimulation and split-belt walking training.

The RAS+SWT group received split-belt treadmill walking training combined with rhythmic auditory stimulation. Each training session lasted 25 min and consisted of a baseline walking phase, an adaptation phase, and a recovery phase. The baseline phase lasted 5 min with both belts running at the same speed to establish a stable initial walking state; during the final 2 min of this phase, rhythmic auditory stimulation was concurrently delivered to familiarize participants with the external rhythm. The adaptation phase lasted 10 min, during which the treadmill belt speed ratio was set to 2:1, with the leg exhibiting the shorter step length placed on the fast belt (10, 17), and rhythmic auditory stimulation was continuously provided. The recovery phase lasted 10 min, during which both belts returned to the same speed and the auditory stimulation was discontinued, to assess the after-effect retention of adaptive improvements. The beat frequency of the rhythmic auditory stimulation was delivered via the “Smart Metronome & Tuner” software. The baseline frequency was determined by calculating the mean cadence from three walking trials at the participant’s comfortable walking speed before the start of the intervention. The beat frequency was then increased by 5 to 10% above the baseline cadence (15). The specific increment was individually adjusted by the supervising therapist: if the participant showed signs of postural instability or difficulty in synchronizing with the rhythm, the increment was set at 5%; if the participant synchronized comfortably while maintaining stable posture, the increment was increased up to 10%. The auditory stimuli were delivered through ceiling-mounted embedded speakers in the gait laboratory, allowing the participant to walk freely on the treadmill without wearing headphones.

The SWT group underwent the same split-belt treadmill training protocol as the RAS+SWT group, including the identical speed ratio (2:1), placement of the shorter-step-length leg on the fast belt, and the same training duration and frequency, but without the provision of rhythmic auditory stimulation during training.

The control group received only the conventional rehabilitation described above, with no split-belt treadmill training or rhythmic auditory stimulation provided.

The total rehabilitation time and intervention components for each group were as follows. All three groups received standard conventional rehabilitation, which consisted of 30 min of manual therapy (including passive joint mobilization, soft tissue manipulation, and stretching) and 20 min of active limb exercises (including strengthening, reaching, and weight-shifting tasks) per session, totaling 50 min per day, five sessions per week. The RAS+SWT and SWT groups each received an additional 25-min treadmill intervention per session, bringing their total daily rehabilitation time to 75 min. The control group received conventional rehabilitation only (50 min/day) without an additional intervention period. Therapist contact time was 55 min/day for the control group (50 min of therapy + 5 min of communication) and 80 min/day for the RAS+SWT and SWT groups (50 min of therapy + 25 min of treadmill supervision + 5 min of communication). Adherence was monitored through daily attendance records; all participants completed 100% of the prescribed sessions.

During all treadmill training sessions, a ceiling-mounted body-weight support system was used to ensure participant safety, with the level of support individually adjusted based on each participant’s standing balance capacity and fatigue level. Participants were permitted to use their usual ankle-foot orthoses during training if routinely used in daily ambulation. Handrails on both sides of the treadmill were available for participants to hold for balance and support when needed. A front handrail was also available, and participants were instructed to use the handrails for light touch support or grasp as necessary, rather than for weight-bearing. Experienced therapists closely supervised each session from beside the treadmill and provided manual assistance only when the participant was at risk of losing balance or falling.

All training sessions were conducted under the full supervision of experienced rehabilitation therapists. Safety assessments, including blood pressure and heart rate measurements and equipment safety checks, were performed before each session. During training, heart rate was continuously monitored using a finger pulse oximeter with automatic recording at 5-min intervals.

### 2.6 Outcome measures

All assessments were completed at two time points: before the intervention (baseline) and after the completion of the intervention (3 weeks). Assessments were performed by the same rehabilitation therapist who was not involved in intervention delivery. The primary outcome measures were spatiotemporal gait parameters, including walking speed, cadence, step length, and the limping index.

All outcome measures were captured using a three-dimensional motion capture system. Participants completed a round-trip walking task on a 6- to 10-meter test walkway, with 3 to 5 valid trials performed per participant. The number of trials was determined by the supervising therapist based on whether the participant had achieved a steady and representative walking pattern. If a consistent and natural gait pattern was observed within the first 3 trials, no additional trials were performed. If variability or inconsistency in gait performance was noted, up to 2 additional trials were conducted to ensure a representative sample. From each trial, three complete gait cycles were extracted, and the average of these three cycles was used to represent that trial. The data were then averaged across the 3 to 5 trials to obtain a single representative value for each participant. Data were acquired using the Vicon Nexus 2.15 three-dimensional gait analysis system, which provides high-precision spatiotemporal measurement and is widely recognized as the gold standard for quantitative gait analysis. Prior to testing, retroreflective markers were placed on the participants’ lower limbs at the following anatomical landmarks: bilateral anterior superior iliac spines, posterior superior iliac spines, greater trochanters, mid-anterior thighs, lateral femoral epicondyles, medial femoral epicondyles, mid-anterior shanks, lateral malleoli, medial malleoli, first, second, and fifth metatarsal heads, and heels.

During overground walking assessments, participants were allowed to use their usual assistive devices and/or ankle-foot orthoses if routinely used. One therapist walked beside the participant during testing for safety but did not provide physical assistance unless the participant was at risk of falling. Participants were given sufficient rest between trials to avoid fatigue.

Overall walking speed and cadence were derived from the Vicon Nexus Plug-in Gait model. For each participant, side-specific values were first calculated for the affected and unaffected limbs, and the overall walking speed and cadence were then computed as the average of the two side-specific values. Specifically, side-specific cadence was calculated as the number of steps taken by the ipsilateral limb per minute, and side-specific walking speed was calculated as stride length divided by stride time for the ipsilateral limb. The overall values were used as the primary outcomes for walking speed and cadence in the present study. Step length was retained as a side-specific outcome, as affected-side and unaffected-side step lengths differed substantially, and this asymmetry is the primary therapeutic target of split-belt treadmill training.

The limping index was calculated using the following formula to quantify gait symmetry at each time point:

$$\text{Limping Index}=\frac{|\text{Step Lengt}h_{\text{affected}}-\text{Step Lengt}h_{\text{unaffected}}|}{\text{Step Lengt}h_{\text{affected}}+\text{Step Lengt}h_{\text{unaffected}}}$$

The index ranges from 0 to 1, with a value closer to 0 indicating greater gait symmetry. This index is an absolute measure of interlimb asymmetry, as the numerator uses an absolute difference.

### 2.7 Statistical analysis

Statistical analysis was performed using R software (version 4.4.2). All statistical tests were two-sided, with the significance level set at α = 0.05.

Baseline characteristics of the participants were first analyzed. Continuous variables were expressed as mean ± standard deviation (Mean ± SD). Comparisons among the three groups were conducted using one-way analysis of variance (ANOVA) under the assumption of homogeneity of variances; when the overall test was significant, *post hoc* pairwise comparisons were performed with Bonferroni correction for multiple comparisons. Categorical variables were expressed as frequencies and percentages, and between-group comparisons were performed using the chi-square test.

Outcome measures were analyzed using linear mixed-effects models (20). The model included time (baseline/post-intervention), group (control group/SWT group/RAS+SWT group), and their interaction (time × group) as fixed effects, with participant as a random effect (random intercept) to account for within-subject correlations arising from repeated measures. Time was coded as 0 for baseline and 1 for post-intervention, and the control group served as the reference level for the group variable. The primary interest was the time × group interaction term, which tested whether the magnitude of change from pre- to post-intervention differed across groups.

To select the optimal model, two nested models were constructed and compared. Model 1 was the base model, containing time, group, and their interaction. Model 2 extended Model 1 by additionally including covariates (sex, age, BMI, stroke type, MMSE score, and modified Barthel Index). Model fit was compared using the difference in the Akaike information criterion (AIC), the difference in the Bayesian information criterion (BIC), and the likelihood ratio test (21), and the optimal model was thereby determined for primary outcome inference.

When the time × group interaction was statistically significant (*p* < 0.05), simple effects analysis was further conducted to identify the specific sources of the interaction effect. The Tukey method was used for multiple comparison correction. To further quantify the practical significance and clinical importance of between-group differences, effect size analysis was performed. The change score for each outcome measure was calculated as Δ = Post – Pre, and effect sizes were computed based on these change scores. Hedges’ g was used as the standardized effect size measure to correct for small-sample bias. Effect sizes were interpreted according to Cohen’s benchmarks: |g| ≥ 0.8 was considered a large effect, 0.5 ≤ |g| < 0.8 a medium effect, and |g| < 0.5 a small effect. The 95% confidence intervals reported for between-group comparisons were not adjusted for multiple comparisons; these analyses were exploratory in nature, and each outcome was pre-specified with an independent *a priori* hypothesis.

## 3 Results

### 3.1 Participant screening and baseline characteristics

The participant screening process is illustrated in Figure 2. A total of 50 patients with post-stroke hemiparesis were screened for this study, of whom 15 did not meet the inclusion criteria, 3 declined to participate, and 2 were excluded for other reasons. Ultimately, 30 participants were enrolled and randomly assigned to the control group (*n* = 11), the SWT group (*n* = 8), and the RAS+SWT group (*n* = 11). All participants completed the 3-week intervention and assessments, with no dropouts or withdrawals.

**Figure 2:**
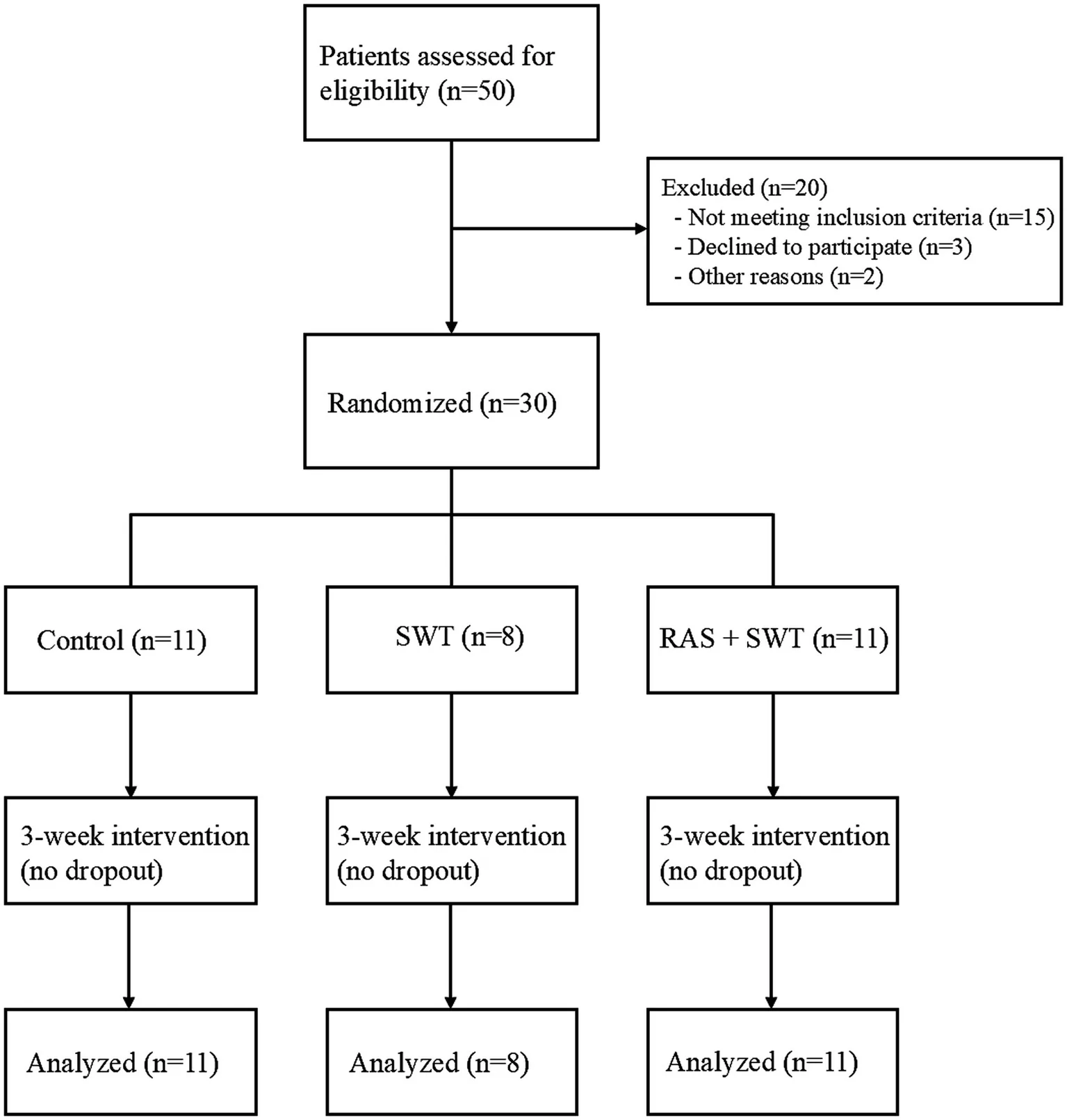
Flow diagram of participant enrollment.

The baseline demographic and clinical characteristics of the three groups are presented in Table 1. No statistically significant differences were observed among the groups in age, sex distribution, time since stroke onset, stroke type, side of hemiparesis, mRS score, BMI, MMSE score, or modified Barthel Index (all *p* > 0.05), indicating that the three groups were comparable at baseline. All participants had a FAC score of 1 according to our institutional assessment, and the conservative nature of this assessment is described in the Methods section. Standing balance level showed a trend toward a between-group difference but did not reach statistical significance (*p* = 0.061).

**Table 1:** Baseline characteristics of participants.

Characteristic	Control group (*n* = 11)	SWT group (*n* = 8)	RAS+SWT group (*n* = 11)	*p* value
Age	47.55 ± 10.26	45.25 ± 8.96	40.41 ± 7.68	0.170
Sex (male/female), *n*	9/2	7/1	10/1	0.819
Time since stroke onset (days)	75.64 ± 28.32	82.38 ± 27.68	91.64 ± 36.17	0.494
Stroke type (hemorrhagic/ischemic), *n*	7/4	7/1	8/3	0.509
Hemiparetic side (left/right), *n*	6/5	6/2	4/7	0.248
Standing balance grade (2/3), *n*	5/6	0/8	2/9	0.061
mRS (3/4), *n*	6/5	5/3	6/5	0.930
FAC = 1, *n*	11	8	11	-
BMI (kg/m^2^)	24.17 ± 3.00	23.18 ± 1.62	25.89 ± 3.16	0.114
MMSE (score)	24.73 ± 5.00	27.38 ± 4.27	27.91 ± 2.84	0.180
Modified Barthel index	75.82 ± 11.31	81.13 ± 4.55	78.82 ± 7.59	0.415

Data are presented as mean ± standard deviation or as counts. Standing balance level was graded according to the Chinese Stroke Rehabilitation Treatment Guidelines: Grade 1, static balance; Grade 2, automatic dynamic balance; Grade 3, dynamic balance under perturbation. All participants had a FAC score of 1, as the FAC assessment in our center tends to be conservative and patients who were able to walk slowly with intermittent assistance were rated as FAC 1. SWT, split-belt walking training; RAS, rhythmic auditory stimulation; RAS+SWT, combined rhythmic auditory stimulation and split-belt walking training. BMI, body mass index; MMSE, Mini-Mental State Examination; mRS, modified Rankin Scale; FAC, Functional Ambulation Category.

### 3.2 Descriptive statistics of spatiotemporal gait parameters

The descriptive statistics of the spatiotemporal gait parameters for each group before and after the intervention are presented in Table 2 and Figure 3. Overall, the RAS+SWT group demonstrated a more pronounced trend toward improvement compared with the control group in walking speed, cadence, step length, and the limping index. In the RAS+SWT group, walking speed increased from 0.37 ± 0.14 m/s to 0.54 ± 0.23 m/s; cadence increased from 73.3 ± 14.5 steps/min to 84.5 ± 17.9 steps/min; and affected-side step length increased from 0.32 ± 0.08 m to 0.39 ± 0.10 m. The limping index decreased from 0.22 ± 0.26 to 0.11 ± 0.17 in the RAS+SWT group. The SWT group also showed a trend toward improvement across all parameters, although the magnitude of improvement was smaller than that observed in the RAS+SWT group. The control group exhibited minimal changes in all parameters from pre- to post-intervention.

**Table 2:** Descriptive statistics of spatiotemporal gait parameters of participants.

Group	Time point	Walking speed (m/s)	Cadence (steps/min)	Step length—unaffected (m)	Step length—affected (m)	Limping index
Control	Pre	0.40 ± 0.23	72.7 ± 19.7	0.29 ± 0.13	0.34 ± 0.13	0.42 ± 0.18
	Post	0.41 ± 0.22	74.6 ± 20.7	0.28 ± 0.11	0.33 ± 0.12	0.37 ± 0.18
SWT	Pre	0.36 ± 0.16	71.5 ± 9.5	0.26 ± 0.11	0.34 ± 0.14	0.34 ± 0.26
	Post	0.42 ± 0.17	78.3 ± 8.8	0.41 ± 0.35	0.36 ± 0.14	0.32 ± 0.15
RAS+SWT	Pre	0.37 ± 0.14	73.3 ± 14.5	0.28 ± 0.08	0.32 ± 0.08	0.22 ± 0.26
	Post	0.54 ± 0.23	84.5 ± 17.9	0.35 ± 0.10	0.39 ± 0.10	0.11 ± 0.17

SWT, split-belt walking training; RAS, rhythmic auditory stimulation; RAS+SWT, Combined rhythmic auditory stimulation and split-belt walking training.

**Figure 3:**
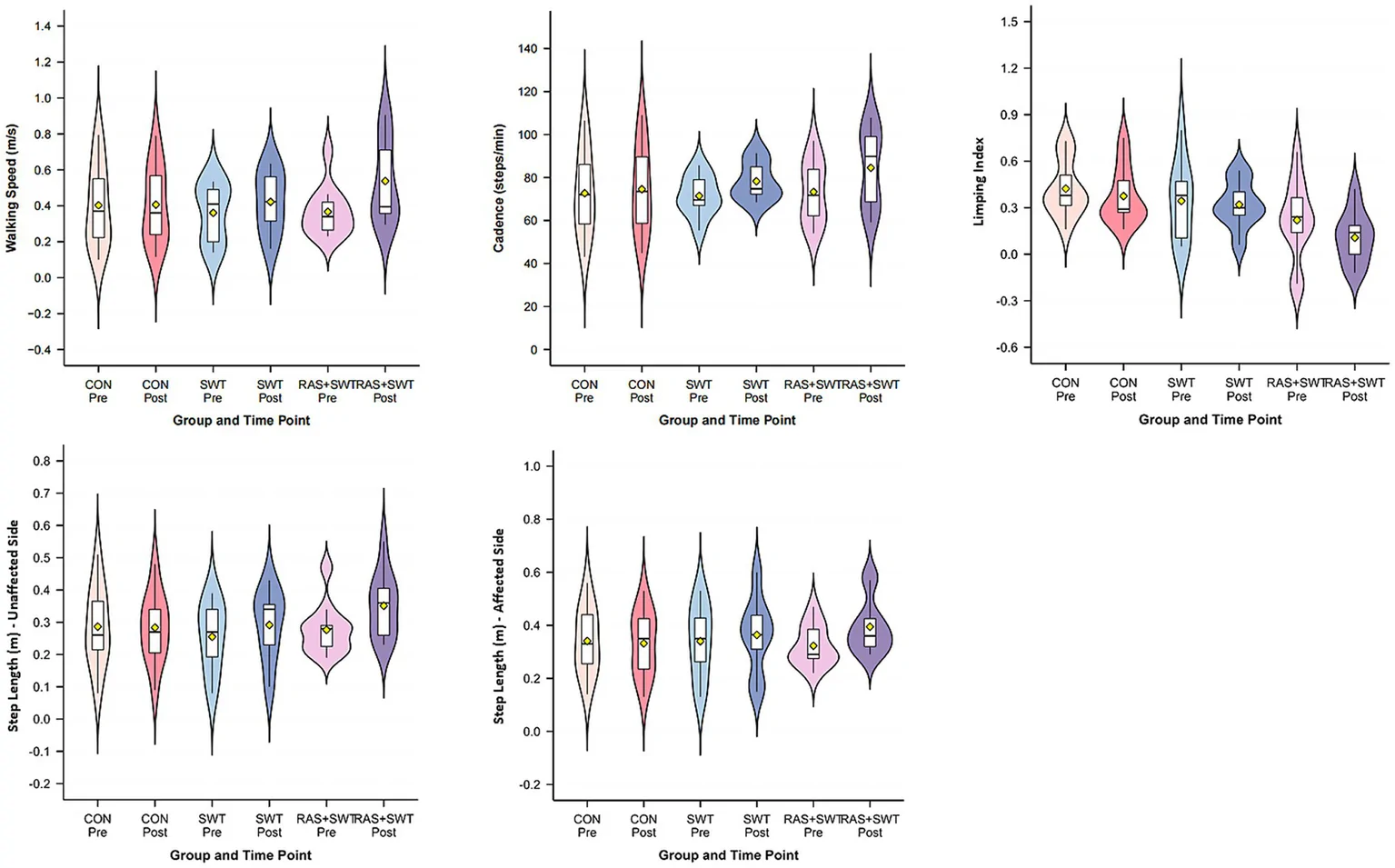
Violin plots showing the distribution of values for each outcome measure. SWT, split-belt walking training; RAS, rhythmic auditory stimulation; RAS+SWT, Combined rhythmic auditory stimulation and split-belt walking training.

### 3.3 Results of linear mixed-effects model analysis

#### 3.3.1 Model comparison and selection

To identify the optimal model, the goodness-of-fit of the base model (Model 1) and the model incorporating covariates (Model 2) was compared for each outcome measure. The likelihood ratio test results indicated that, for the majority of outcome measures, with the exception of affected-side step length, Model 2 did not yield a significant improvement in goodness-of-fit over Model 1 (*p* > 0.05). Moreover, the AIC and BIC values of Model 1 were lower than those of Model 2, suggesting that the inclusion of covariates did not confer a consistent advantage in model fit. Accordingly, Model 1 was adopted as the primary analytical model in this study.

#### 3.3.2 Walking speed

A significant time × group interaction effect was observed for walking speed (*F* = 10.68, *p* < 0.001), indicating that the improvement in walking speed differed across intervention modalities. Simple effects analysis revealed that in the RAS+SWT group, walking speed increased significantly from pre- to post-intervention (β = 0.171, *p* < 0.001), with a Hedges’ g of 1.741 compared with the control group. In the SWT group, the improvement in walking speed did not reach statistical significance after correction for multiple comparisons (β = 0.061, *p* = 0.054), with a Hedges’ g of 1.175 compared with the control group. No statistically significant pre–post differences were observed in the control group. Interaction plots for walking speed are presented in Figure 4. Between-group effect sizes are shown in Table 3.

**Figure 4:**
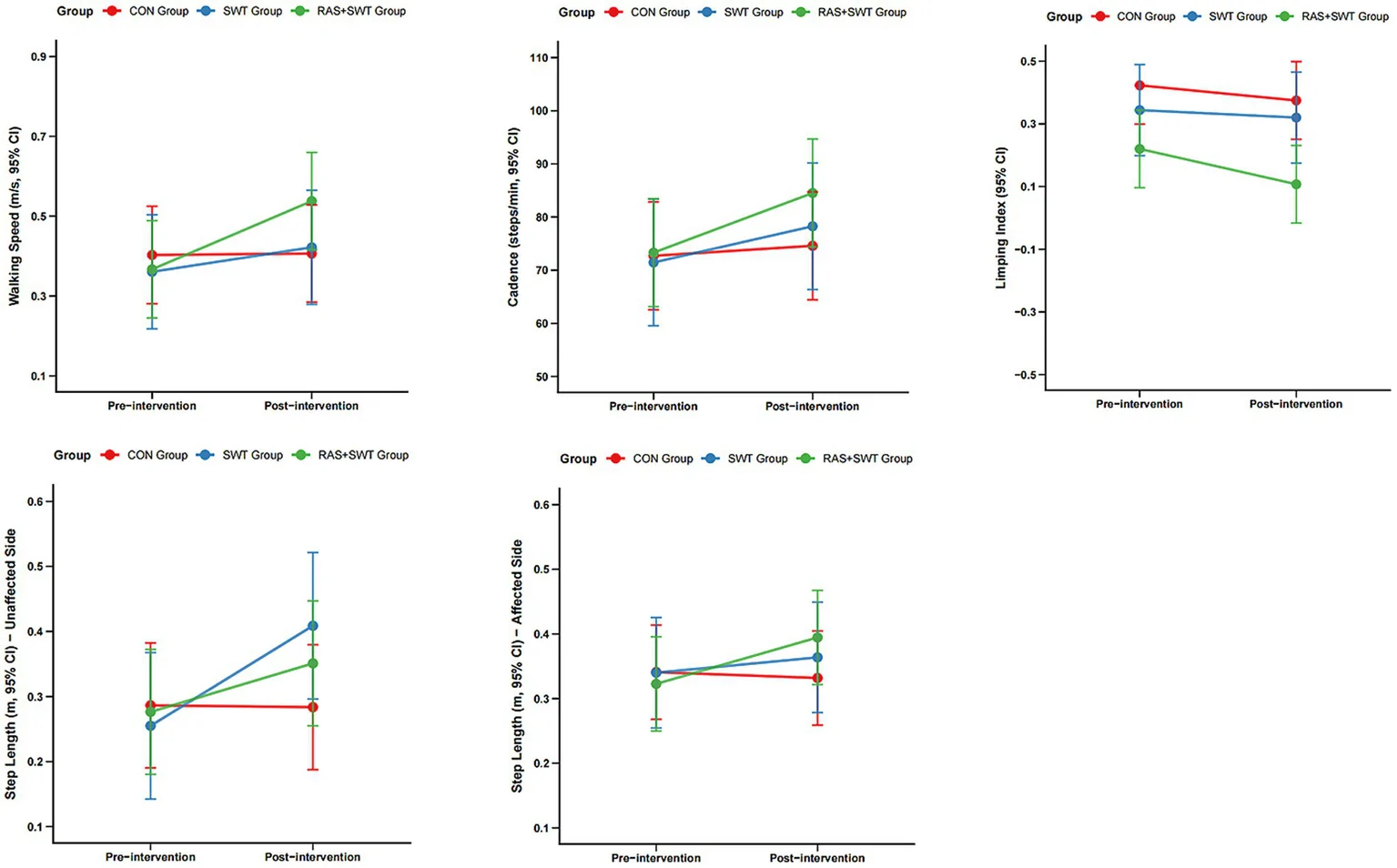
Time × group interaction plots for spatiotemporal gait parameters. SWT, split-belt walking training; RAS, rhythmic auditory stimulation; RAS+SWT, combined rhythmic auditory stimulation and split-belt walking training.

**Table 3:** Between-group comparisons of changes in spatiotemporal gait parameters.

Outcome	Comparison	Mean difference (95% CI)	Hedges’ g
Walking speed	RAS+SWT vs. CON	0.167 [0.090, 0.245]	1.741
	RAS+SWT vs. SWT	0.110 [0.022, 0.197]	0.988
	SWT vs. CON	0.058 [0.010, 0.105]	1.175
Cadence	RAS+SWT vs. CON	9.340 [4.456, 14.223]	1.538
	RAS+SWT vs. SWT	4.407 [−3.636, 12.451]	0.488
	SWT vs. CON	4.932 [−1.615, 11.479]	0.768
Step length (unaffected)	RAS+SWT vs. CON	0.077 [0.039, 0.116]	1.620
	RAS+SWT vs. SWT	−0.079 [−0.344, 0.185]	−0.279
	SWT vs. CON	0.156 [−0.107, 0.420]	0.555
Step length (affected)	RAS+SWT vs. CON	0.081 [0.035, 0.127]	1.413
	RAS+SWT vs. SWT	0.048 [−0.009, 0.105]	0.713
	SWT vs. CON	0.033 [−0.005, 0.070]	0.805
Limping index	RAS+SWT vs. CON	−0.065 [−0.258, 0.129]	−0.279
	RAS+SWT vs. SWT	−0.089 [−0.290, 0.112]	−0.393
	SWT vs. CON	0.024 [−0.149, 0.198]	0.128

The 95% confidence intervals for between-group comparisons were not adjusted for multiple comparisons, as these analyses were exploratory in nature; results close to the boundary should therefore be interpreted with caution. SWT, split-belt walking training; RAS, rhythmic auditory stimulation; RAS+SWT, Combined rhythmic auditory stimulation and split-belt walking training.

#### 3.3.3 Cadence

A significant time × group interaction effect was observed for cadence (*F* = 5.01, *p* = 0.014). Simple effects analysis showed that in the RAS+SWT group, cadence increased significantly from pre- to post-intervention (β = 11.22, *p* < 0.001), with a Hedges’ g of 1.538 compared with the control group. In the SWT group, cadence also improved significantly from pre- to post-intervention (β = 6.81, *p* = 0.010), with a Hedges’ g of 0.768 compared with the control group. No statistically significant pre–post differences in cadence were found in the control group. Between-group effect sizes are presented in Table 3.

#### 3.3.4 Step length

For unaffected-side step length, the time × group interaction effect was not significant (*F* = 1.47, *p* = 0.247), whereas the main effect of time was significant (*F* = 4.27, *p* = 0.048). For affected-side step length, the time × group interaction effect was significant (*F* = 6.21, *p* = 0.006). Simple effects analysis demonstrated that in the RAS+SWT group, affected-side step length increased significantly from pre- to post-intervention (β = 0.072, *p* < 0.001), with a Hedges’ g of 1.413 compared with the control group and a Hedges’ g of 0.713 compared with the SWT group. No statistically significant pre–post differences in affected-side step length were observed in the SWT group or the control group, with a Hedges’ g of 0.805 between the SWT and control groups. Between-group effect sizes are shown in Table 3.

#### 3.3.5 Limping index

For the limping index, the time × group interaction effect was not significant (*F* = 0.430, *p* = 0.655), whereas a significant main effect of group was found (*F* = 5.41, *p* = 0.011). Between-group comparisons revealed that post-intervention, the RAS+SWT group had a significantly lower limping index than the control group (β = −0.267, *p* = 0.010), with a Hedges’ g of −0.279. The Hedges’ g for the comparison between the RAS+SWT group and the SWT group was −0.393. Between-group effect sizes are presented in Table 3.

## 4 Discussion

This randomized controlled trial, conducted over a 3-week intervention period, found that rhythmic auditory stimulation combined with split-belt walking training was more effective than conventional rehabilitation in improving walking capacity in patients with post-stroke hemiparesis. Compared with SWT alone, the combined intervention yielded greater improvements in walking speed, while no significant between-group differences were observed for cadence or step length. Specifically, the RAS+SWT group demonstrated superior improvements over conventional rehabilitation across multiple spatiotemporal parameters, including walking speed, cadence, and affected-side step length. These findings indicate that the integration of split-belt walking training, which targets spatial adaptation, with rhythmic auditory stimulation, which targets temporal rhythmicity, may produce a combined effect, offering a novel intervention strategy for gait rehabilitation in patients with post-stroke hemiparesis.

### 4.1 Analysis of intervention effects

Regarding split-belt walking training, prior research has rather consistently demonstrated its efficacy in ameliorating spatial gait asymmetry. Reisman et al. (22) were among the first to show in patients with stroke that repeated split-belt walking training could significantly reduce step length asymmetry, and that this improvement could be partially retained after the perturbation was removed—a phenomenon known as the after-effect. Lewek et al. (18) further noted that error augmentation training using a split-belt treadmill could effectively reduce step length asymmetry, yet their study also suggested that error augmentation was not superior to error minimization, and that other factors, such as verbal feedback, might also serve as core drivers of step length improvement. A more recent study by Wang et al. (19) reported similar findings, showing that four weeks of split-belt walking training significantly improved spatial gait symmetry and lower limb function. In the present study, the SWT group yielded a Hedges’ g of 0.805 for affected-side step length compared with the control group, an effect size comparable to those reported in the aforementioned studies, further corroborating the effectiveness of split-belt walking training in improving spatial asymmetry. However, the improvement in affected-side step length in the SWT group did not reach statistical significance, whereas that in the RAS+SWT group did. When interpreted alongside the findings of Lewek et al. (18), this suggests that RAS in the present study may have played a role analogous to that of verbal feedback as an augmenting cue. Although SWT alone produced a medium-to-large effect size, its effect was not statistically robust; the addition of RAS may have enhanced the sensorimotor adaptation process by providing a stable temporal reference frame, thereby rendering the improvement in affected-side step length statistically detectable.

With respect to rhythmic auditory stimulation, previous research has demonstrated that RAS can effectively improve temporal gait parameters in patients with stroke, including cadence, gait rhythmicity, and temporal symmetry (15). In the present study, the RAS+SWT group exhibited superior improvements in temporally related measures such as cadence and walking speed compared with the control group, with effect sizes reaching a large magnitude.

To evaluate the clinical meaningfulness of these findings, we compared the observed improvements with established minimal clinically important difference (MCID) thresholds for gait speed in stroke populations. Tilson et al. reported an MCID of 0.16 m/s for comfortable gait speed in subacute stroke patients (23), and Bohannon et al. reported an MCID of 0.13 m/s in patients undergoing inpatient rehabilitation (24). In our study, the RAS+SWT group showed a mean improvement of 0.17 m/s in walking speed, which exceeds both of these thresholds. This suggests that the observed improvement is not only statistically significant but also clinically meaningful.

Notably, the difference in cadence improvement between the RAS+SWT group and the SWT group was relatively small, with effect sizes in the small-to-medium range, suggesting that split-belt walking training itself may exert a certain perturbing effect on cadence, likely attributable to the direct impact of the inter-limb belt speed difference on temporal gait parameters.

Importantly, although the cadence improvements did not differ substantially between the RAS+SWT and SWT groups, the walking speed improvement was significantly greater in the RAS+SWT group than in the SWT group. This finding highlights a unique advantage of the combined intervention: by providing stable rhythmic cues, RAS creates a favorable temporal control framework (25), which may enable patients to more effectively translate the spatial adjustments acquired during split-belt walking training into actual step length gains (26), thereby producing a combined effect. When SWT is applied in isolation, patients may achieve some degree of step length improvement, but the absence of rhythmic guidance may render their spatial adaptation insufficiently stable or incompletely expressed. Cherry-Allen et al. (27) found that in patients with stroke, the provision of tandem auditory feedback following split-belt walking adaptation produced more durable improvements in step length symmetry, and the majority of participants adopted a pattern of lengthening the shorter step. This finding aligns with the results of the present study and further supports the additive benefit of combining RAS and SWT.

For the limping index, the time-by-group interaction was not significant (*p* = 0.655), indicating that the three groups did not differ in the magnitude of change over time. Nevertheless, a significant main effect of group was observed (*p* = 0.011), with the RAS+SWT group showing a lower value than the control group at post-intervention; however, a certain difference already existed between the two groups at baseline. This baseline difference may be attributable to sampling variation during the randomization process. Accordingly, the between-group difference at post-intervention should be interpreted with caution, as it may reflect both the true effect of the combined intervention and the persistence of a baseline advantage. Previous studies have reported inconsistent findings regarding gait symmetry: some have found that split-belt walking training significantly improves symmetry (12), whereas others have not observed significant improvements immediately post-intervention but have detected significant gains at follow-up (10), a discrepancy that may be attributable to the after-effect enhancement of SWT. In the present study, the change in the limping index in the RAS+SWT group did not differ significantly from that in the SWT group, suggesting that within the 3-week intervention period, the effect of the combined intervention on symmetry may not yet have fully manifested. Future studies with longer intervention periods are warranted to verify whether a significant effect on gait symmetry can be detected.

### 4.2 Clinical application and protocol optimization

The RAS combined with SWT protocol adopted in this study holds considerable promise for clinical translation. Split-belt treadmills are already available in large rehabilitation facilities, and RAS can be implemented using only a smartphone (28). Moreover, this combined intervention is of moderate intensity and carries a low risk of adverse events; indeed, no serious adverse events occurred throughout the entire intervention period in this study, indicating a favorable safety profile. The patients enrolled in this study were at a moderate-to-severe stage of walking dysfunction (FAC = 1, modified Rankin Scale score 3–4) yet had already acquired basic standing and weight-bearing capacity. These patients characteristically exhibit pronounced rhythm abnormalities and gait asymmetry (29), making them an ideal target population for this combined intervention. In terms of the phase of rehabilitation, the participants in this study were predominantly in the subacute stage (mean time since stroke onset: 83.3 ± 31.01 days; range: 36–155 days), with some approaching the chronic stage. This aligns with the recommendation from a previous review that gait pattern optimization training is appropriate during this phase (30). Unlike conventional aerobic exercise, which primarily enhances walking endurance, the advantage of this combined intervention lies in its targeted optimization of the gait pattern itself; the two strategies can therefore be complementary in clinical practice.

In addition, the plateau effect observed in cadence improvement in this study provides a reference for the individualized setting of RAS tempo. An excessively small increase in tempo may fail to induce a sufficient rhythm entrainment effect, whereas an excessively large increase may compromise postural stability (31). Responses to rhythmic cues vary across patients: some may benefit from a larger increase in tempo, whereas others require a more conservative setting (31). This suggests that, in clinical practice, the RAS tempo should be dynamically adjusted based on patients’ real-time responses rather than being uniformly fixed at a predetermined increment. Furthermore, the finding that the limping index showed a trend toward improvement but did not reach statistical significance after the 3-week intervention provides direction for optimizing the intervention dose. Drawing on evidence regarding exercise dose, a higher total training volume may be necessary to consolidate improvements in gait symmetry (30). In the present study, the total duration of the 3-week intervention was approximately 375 min, suggesting that future clinical protocols could consider extending the intervention period or increasing the duration of individual sessions to achieve more pronounced improvements in symmetry.

In clinical practice, this combined intervention should serve as a complement to, rather than a replacement for, conventional rehabilitation (30). Therapists can integrate it with conventional aerobic training and strength training in a tailored manner based on the specific functional deficits of each patient (30). It is recommended that the intervention be initiated once patients have acquired basic weight-bearing capacity, and that reinforcement exercises be conducted during overground walking following the training sessions to promote the transfer of training gains to everyday environments. Therapists should also closely monitor patients’ adaptive responses to the RAS tempo; should postural instability or increased fatigue occur, the tempo should be promptly reduced or returned to baseline levels.

### 4.3 Limitations and future research directions

Several limitations of this study should be acknowledged. First, the sample size was relatively modest (*n* = 30), primarily constrained by the inpatient capacity of a single rehabilitation center during the study period. Although the total sample size met the *a priori* requirement (30 participants), the SWT group (*n* = 8) was smaller than the other two groups (*n* = 11 each) due to the randomization process. This imbalance may have reduced statistical power for between-group comparisons involving the SWT group, particularly for outcomes with marginal significance (e.g., walking speed, *p* = 0.054). Second, despite the observed effect sizes, the modest sample size warrants cautious interpretation of the findings. Third, the intervention period was relatively short (3 weeks) without long-term follow-up, primarily due to the limited length of stay of patients in the rehabilitation hospital. Consequently, the durability of the improvements remains unknown. Fourth, a major limitation of this study is the absence of a time- and attention-matched control group. The RAS+SWT and SWT groups each received an additional 25-min treadmill intervention per session, five sessions per week, beyond standard care, whereas the control group received conventional rehabilitation only (75 min/day vs. 50 min/day). Consequently, the observed between-group differences may partly reflect nonspecific factors such as increased total walking practice, additional therapist contact, higher treatment intensity, or enhanced participant motivation, rather than the specific effects of SWT or RAS alone. We acknowledge that this design limits our ability to isolate the specific contribution of the combined intervention from the effects of additional therapeutic contact. However, the comparison between RAS+SWT and SWT was largely matched for total training time and treadmill exposure (both groups received 75 min/day with 25 min on the treadmill), allowing us to isolate the specific contribution of RAS. The RAS+SWT vs. control comparison, while not time-matched, addresses a clinically relevant question: whether adding this combined intervention to routine care yields incremental benefits. This mirrors real-world clinical practice, where targeted interventions are added to, rather than replacing, standard rehabilitation. Fifth, the FAC assessment in our center may be more conservative than in other centers. Although all participants had a FAC score of 1, their actual functional walking ability was better than the conventional interpretation of FAC 1 would suggest, as supported by their Barthel Index (75–81), standing balance grade (2–3), and ability to complete the training protocol. This may limit the generalizability of our findings, and future studies should use standardized FAC assessment protocols across centers. Sixth, we did not systematically classify participants according to specific clinical motor dysfunction patterns (e.g., equinovarus foot, stiff knee, or flexed hip), as described by Mayer et al. (32). This may have introduced heterogeneity in intervention responses, as different deformity patterns could influence the effects of split-belt treadmill training and rhythmic auditory stimulation. Future studies should incorporate detailed clinical phenotyping to identify which subgroups benefit most from the combined intervention. Seventh, detailed kinematic analysis of lower limb joint angles was not included as a pre-specified outcome in this study. Future studies incorporating kinematic analysis could provide additional insights into the movement strategies underlying the observed improvements. These limitations notwithstanding, the present findings should be considered preliminary, and large-sample, multicenter trials with extended follow-up are needed to confirm and extend these results.

Future research could proceed in several directions. Large-sample, multicenter randomized controlled trials are warranted to validate the preliminary findings of the present study and to explore differential responses to the combined intervention across patient subgroups (e.g., different sides of hemiparesis, varying disease durations, and different degrees of baseline asymmetry), thereby helping to identify the population most likely to benefit from this protocol. Extending the intervention period and incorporating long-term follow-up time points (e.g., 3 months and 6 months) would allow for the assessment of the durability and clinical significance of the intervention effects, particularly the long-term retention of symmetry improvements. The optimization of individualized intervention prescriptions represents another important avenue; future studies could investigate the suitability of different RAS tempo increments (e.g., 5%, 10%, and 15%) and different SWT speed ratios (e.g., 2:1, 3:1, and 4:1) for different patients. Moreover, incorporating assessments of real-world walking capacity and adopting outcome measures with greater ecological validity (e.g., daily step count monitored via wearable devices) would help verify the transfer of intervention effects to everyday life. Finally, exploring the neural mechanisms underlying the combined intervention—for instance, using functional near-infrared spectroscopy to evaluate changes in cortical activation patterns before and after training, or using transcranial magnetic stimulation to assess alterations in corticospinal excitability—could reveal the neural-level interplay between auditory–motor coupling and sensorimotor adaptation.

## 5 Conclusion

This randomized controlled trial found that rhythmic auditory stimulation combined with split-belt walking training was more effective than conventional rehabilitation in improving walking capacity in patients with post-stroke hemiparesis. Compared with SWT alone, the combined intervention yielded greater improvements in walking speed, while no significant between-group differences were observed for cadence or step length. The advantages of the combined intervention over conventional rehabilitation were primarily manifested in improvements in spatiotemporal parameters, including walking speed, cadence, and affected-side step length. For the limping index, the time-by-group interaction was not statistically significant, indicating that the three groups did not differ in the magnitude of change over time. The contribution of RAS to cadence improvement appeared to overlap with the spontaneous adjustment effects induced by SWT, as the between-group difference in cadence improvement did not reach statistical significance. In addition, this combined intervention protocol demonstrates favorable clinical feasibility and safety, and is suitable for patients with post-stroke hemiparesis who are in the subacute stage, have acquired a certain level of walking ability, yet present with rhythm abnormalities or gait asymmetry. It is recommended that the RAS tempo and intervention dose be individualized according to the patient’s specific functional status and delivered as an adjunct to conventional rehabilitation. However, this study has several limitations, including a relatively small sample size, a short intervention period, and the absence of long-term follow-up. Future studies should recruit larger samples, extend the intervention duration, and incorporate follow-up time points to verify the long-term effects of the combined intervention.

## Data Availability

The raw data supporting the conclusions of this article will be made available by the authors, without undue reservation.
